# Protective effects of gallic acid and SGK1 inhibitor on oxidative stress and cardiac damage in an isolated heart model of ischemia/reperfusion injury in rats

**DOI:** 10.22038/IJBMS.2023.68045.14874

**Published:** 2023-03

**Authors:** Faramarz Souri, Mohammad Badavi, Mahin Dianat, Seyed Ali Mard, Alireza Sarkaki

**Affiliations:** 1 Department of Physiology, School of Medicine, Ahvaz Jundishapur University of Medical Sciences, Ahvaz, Iran; 2 Persian Gulf Physiology Research Center, Medical Basic Sciences Research Institute, Ahvaz Jundishapur University of Medical Sciences, Ahvaz, Iran

**Keywords:** Gallic acid, GSK650394, Heart, Oxidative stress, Reperfusion injury, Serum-glucocorticoid - regulated kinase 1

## Abstract

**Objective(s):**

Oxidative stress and serum and glucocorticoid-induced Kinase 1 gene (SGK1) perform a central role in the consequences of ischemia in the heart. This research aimed to investigate the effect of coadministration of gallic acid and the GSK650394 (as SGK1 gene inhibitor) on the ischemic complications of a rat model of cardiac ischemia/reperfusion (I/R) injury.

**Materials and Methods:**

Sixty male Wistar rats were divided into 6 groups with or without pretreatment with gallic acid for 10 days. After that, the heart was isolated and perfused with Krebs-Henseleit solution. A 30 min of ischemia was performed followed by a 60 min reperfusion. In 2 groups, GSK650394 was infused 5 min before ischemia induction. Ten minutes after reperfusion commencement, cardiac marker enzyme (CK-MB, LDH, and cTn-I) activities were measured in the cardiac perfusate. At the end of reperfusion, the activity of anti-oxidant enzymes (Catalase, Superoxide dismutase, and Glutathione peroxidase), lipid peroxidation (MDA), total anti-oxidant capacity (TAC), intracellular reactive oxygen species (ROS), infarct size, and SGK1 gene expression were measured in the heart tissue.

**Results:**

The results indicated that dual therapy with both drugs significantly improved endogenous anti-oxidant enzyme activity and TAC more than each drug alone. However, the heart marker enzymes (CK-MB, LDH, and cTn-I), MDA, ROS, infarct size, and SGK1 gene expression were reduced significantly compared with the ischemic group.

**Conclusion:**

The results of this study suggest that concomitant administration of both drugs in the case of cardiac I/R injury may have a more beneficial effect than each one alone.

## Introduction

Myocardial damage, an essential pathological change of coronary artery disorder because of ischemia/reperfusion (I/R), is a fundamental problem that desires attention (1). Myocardial ischemia occurs when coronary blood flow to the myocardium is obstructed. Subsequently, re-establishing the blood flow will improve cardiac function and decrease infarct size. Although reperfusion improves cardiac function and infarct volume, after a while it increases oxygen free radicals (2). Raised oxidative stress is also produced during reperfusion of the myocardium after infarction due to the concurrent rapid release of free radicals (3). In the heart, reactive oxygen species (ROS) formation can be induced by cytokines and growth factors. That is one of the features that occur with a sudden change in the integrity of heart cells and a complete loss of cardiac contractile function associated with severe ultra-structural damage (4). The interaction between cell membrane lipids and ROS leads to lipid peroxidation and production of a cytotoxic product called Malondialdehyde (MDA), which leads to membrane destruction, myocardial cell injury, heart failure, and irreversible tissue damage. These proceedings are associated with a decrease in endogenous anti-oxidant compounds, including Catalase (CAT), Glutathione peroxidase (GPx), Superoxide dismutase (SOD), and glutathione system (GSH) (5). Also, raised cardiac enzymes, particularly lactate dehydrogenase (LDH), creatine kinase˗MB (CK˗MB), and cardiac troponin-I (cTn-I) indicate myocardial cell damage and necrosis (6, 7).

Serum and glucocorticoid˗induced Kinase 1 (SGK1) is a transcriptionally induced Serine˗Threonine Kinase that is perhaps involved in ischemia/reperfusion (I/R) and myocardial infarction (8, 9). SGK1 expression is highly variable and regulated by many pathogens and ischemia. SGK1 is expressed everywhere, including in the liver, heart, kidneys, arteries, and other organs (10, 11), and its expression is high in the heart (12). Evidence showed that SGK1 expression is altered in pathological conditions (13). Since SGK1 regulates the activity of transporters and ion channels and affects blood pressure (BP), it is very likely that SGK1 influences stroke outcome (14). Remarkably, microarray data show that its expression in the human brain tends to rise with age. This suggests that SGK1 may also be associated with a high rate of stroke in aging (15).

Gallic acid (3, 4, 5^_^trihydroxy benzoic acid), a natural secondary metabolite with low molecular weight, is found in various plants and is commonly used in medicine. Gallic acid increases the anti-oxidant capacity and the activity of CAT, GPx, and SOD in the heart isolated from alloxan˗induced diabetic mice (16). It also prevents damage to ROS by blocking the opening of mPTP from cell membranes and mitochondria. In addition, gallic acid protects the isolated heart of mice against damage by increasing the capacity of the endogenous anti-oxidant system (17).

As previously mentioned, oxidative stress plays a key role in the consequences of ischemia in the heart (3). Furthermore, SGK1 may be involved in the inflammatory process and cardiac dysfunction in the isolated heart due to I/R injury (18). Since the use of these two compounds alone has good protective effects against ischemic complications in the heart models of I/R injury, this research aimed to evaluate the possible more beneficial cardioprotective effects of SGK1 inhibitor (GSK650394) and gallic acid coadministration on oxidative stress, infarct size, cardiac enzyme markers, and other complications induced by global I/R injury in isolated rat heart.

## Materials and Methods

***Materials***

Xylazine (2%) and HCl ketamine (10%) were received from Alfasan Co (Netherlands). Krebs-Henseleit salts were received from Merck Co (Germany). Gallic acid, heparin, and 2,3,5˗triphenyl tetrazolium chloride (TTC) were received from Sigma (St Louis, MO, USA). GSK650394 (GSK) was purchased from MedChemExpress (USA). Anti-oxidants, TAC, and MDA analysis kits were received from the Sigma Zell Bio Co (Germany). CK˗MB isoenzyme and LDH activity were received from the Pars Azmoon Co (Iran). Cardiac Troponin-I as a specific biomarker of heart damage was measured by a specific kit obtained from Monobind Inc. (Lake Forest, California, USA).

***Animals***

The study was done on 60 male adult Wistar rats (weighing 250–300 g) received from the animal house of Ahvaz Jundishapur University of Medical Sciences one week before the conduction of the experiments and had free access to food and water in a 12:12 hr light^_^dark cycle and controlled temperature (22 ± 2 °C) condition. The protocols of the experiments were approved by the research ethics committee of the Research Center & Experimental Animal House at Ahvaz Jundishapur University of Medical Sciences (ID: IR. AJUMS. ABHC. REC. 1400.032, dated: 2021-05-25). They were randomly divided into 6 groups (10 in each group, Table 1). 

Gallic acid was dissolved in normal saline and administrated (30 mg/kg daily by gavage) for 10 days (19). In those groups that received GSK650394, 5 min before induction of ischemia, the isolated heart was perfused with Krebs-Henseleit solution containing 1 μmol of GSK650394 (20), prepared in DMSO (17). For those groups that did not receive gallic acid or GSK650394, the vehicles of the drugs were administered instead. Ischemia induction was performed by complete closure of the infusion flow for 30 min and then reperfusion for 60 min (17). 

***Heart isolation and ischemia induction***

The animals were anesthetized by injecting xylazine (5 mg/kg) and HCl ketamine (50 mg/kg) intraperitoneally. To prevent blood clots, heparin was injected intraperitoneally (1000 U/kg) (17). The trachea was then cannulated and attached to the rodent’s ventilator (UGO BASILE, model: 7025), to allow artificial breathing room air, after opening the chest and removing the ribs (17). The aorta was cannulated by introduction of a stainless-steel cannula through a cut in its wall and fixed by a suture. The heart was immediately infused with Krebs˗Henseleit solution which consisted of glucose, MgSO_4_, KH_2_PO_4_, NaCl, KCl, NaHCO_3_, and CaCl_2_ (11.1 mM, 1.2 mM, 1.18 mM, 118 mM, 4.75 mM, 25 mM, and 1.75 mM, respectively) equilibrated by 5% CO_2_ and 95% O_2_ at pH of 7.4. The heart was then excised and transferred to a Langendorff device containing Krebs˗Henseleit solution. The isolated heart was constantly perfused with the solution by a peristaltic pump at a constant flow of 7 ± 2 ml.min^-1^ and temperature (37 °C) (21). During the research, the buffer was bubbled with 95% O2-5% CO_2_ to maintain a pH of 7.4. The ischemia was induced by the no flow method for 30 min as mentioned in the previous section and then reperfused for 60 min (17). 

***Anti-oxidant enzymes*** ***′*** ***activity and MDA content***

At the end of reperfusion, 100 mg of cardiac tissue was removed and homogenization was performed in 1 ml of cold phosphate-buffered saline (PBS). Following centrifuging at 4000 rpm for fifteen min, the supernatant was used for measuring MDA, TAC, and the activity of anti-oxidant agents with special kits and reagents (22).

***MDA analysis***

For the lipid peroxidation analysis, the MDA content was measured calorimetrically at 535 nm, according to the manufacturer’s guidelines (Zell Bio GmbH, Germany) (23). 

***SOD activity***

SOD activity was measured at a wavelength of 420 nm using a colorimetric method and according to the manufacturer’s guidelines (Zell Bio GmbH, Germany). The amount of tissue section used to catalyze 1 µmol O^2-^ to H_2_O_2_ and O_2_ in 1 min, becomes decided as a unit of SOD activity (23).

***CAT activity***

CAT activity was assessed colorimetrically at a wavelength of 405 nm according to the manufacturer’s guidelines (Zell Bio GmbH, Germany). The quantity of the tissue section that was used to catalyze 1 µmole of H_2_O_2_ to water and O_2_ in 1 min, becomes decided as a unit of CAT activity (23). 

***GPx activity***

To determine GPx activity, a colorimetric method was performed at a wavelength of 412 nm according to the manufacturer’s guidelines (Zell Bio GmbH, Germany). The quantity of sample used to catalyze 1 μmole of GSH to GSSG for 1 min turned into decided as a unit of GPx activity (23).

***TAC measurement***

To determine TAC, a colorimetric method was performed at a wavelength of 490 nm according to the manufacturer’s guidelines (Zell Bio GmbH, Germany) (23).

***Intracellular ROS assay***

Reactive oxygen species activity within the cells was measured by Cell Bio labs’ OxiSelect™ Intracellular ROS Assay Kit according to the manufacturer’s guidelines. Detection of green fluorescence (excitation: 495 nm; emission: 515 nm) was performed by a plate reader (24).

***Evaluation of myocardial injury***

To assess the ischemic myocardial injury, fluid samples were taken from the heart 10 min after reperfusion to measure cTn-I, LDH, and CK˗MB activity. The measurements were performed by an ELISA reader according to the kit’s instructions (25).

***Infarct size measurement***

At the end of the above-mentioned trials, the hearts were removed from the Langendorff apparatus and placed in the freezer for 2 hr. The incisions were then prepared with a thickness of 2 mm and stained with 2,3,5˗Triphenyl Tetrazolium Chloride (TTC) at 37 ° C for 20 min. Then the tissue was fixed in 10% formalin for 24 hr to measure the size of the infarction. Then, the infarct area was measured using ImageJ software (NIH, Bethesda, MD, USA) by calculating the percentage of infarct area relative to the total slice area bilaterally (26).

***SGK1 gene expression analysis in heart tissue***

The total mRNA of SGK1 and the housekeeping gene, glyceraldehyde-3-phosphate dehydrogenase (GAPDH) was extracted from the frozen heart tissue samples, using an extraction kit (Ampliqon, Denmark). The concentration and purity of the extracted mRNA were determined spectrophotometrically at 260 and 280 nm wavelengths (Eppendorf, Nanodrop Thermo Scientific S.N: D015). Next, the cDNA was synthesized from total mRNA, using a cDNA Synthesis Kit (Ampliqon, Denmark), following the manufacturer’s guidelines. Real˗Time PCR (RT˗PCR) was used to determine SGK1 gene expression in heart tissue using RunMei Q200 apparatus (China). The following particular primers (Bioneer, Daejeon, South Korea) were used in this research: GAPDH, (forward: 5′CAGCCTCAAGATCATCAGCAATG′3 and reverse 3′CATGAGTCCTTCCACGATACCA′5) SGK1, (forward: 5′AATGGCGGAGAGCTGTTCTA′3 and reverse 3′TGCAGATAACCCAAGGCACT′5). To determine the relative quantities of SGK1 gene expression, the comparative cycle of threshold (Ct) method and RunMei QC 3.2 software with arithmetic formulae (2 ^–^^ΔΔ^^Ct^) was used (27).

***Statistical analysis***

The received statistics were analyzed using SPSS version 22 and expressed as mean±SD. The normality of the data was verified using the Kolmogorov˗Smirnov goodness test. Comparisons between groups were made using a one^-^way analysis of variance, followed by Tukey’s multiple comparison test. *P*-values less than 0.05 were considered significant. 

## Results

***Anti-oxidant enzymes and MDA content***

**Figure 1:**
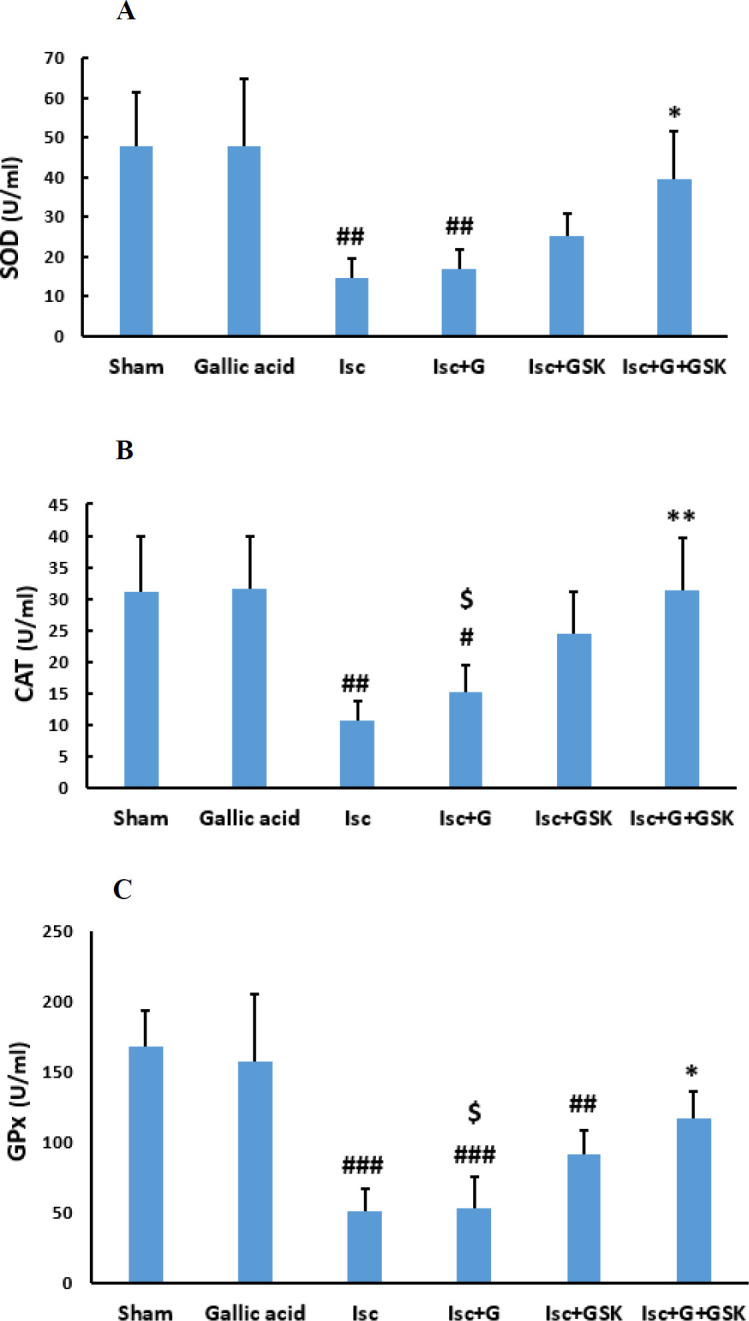
The effects of Gallic acid (G, 30 mg/kg/daily by gavage) and GSK (GSK650394, 1μmol) or their combination on Superoxide dismutase (SOD) (A), Catalase (CAT) (B), and Glutathione peroxidase (GPx) (C) activities in the heart tissue (mean ± SD, n=10) following heart ischemia/reperfusion injury. #* P*<0.05, ## * P*<0.01, and ### * P*<0.001 vs. sham group. * * P*<0.05, and ** P<0.01, vs. Isc (ischemic) group. $ * P*< 0.05 vs. Isc+G+GSK group (One way ANOVA followed by Tukey’s *post hoc* test)

Measurement of the activity of SOD indicated that its activity was significantly reduced in the ischemic (Isc) group compared with the sham group (*P*<0.01). However, compared with the Isc group, treatment with gallic acid or GSK650394 alone did not change its activity significantly (Figure 1A). However, by concomitant therapy with these two compounds (Isc+G+GSK group) its activity increased significantly compared with the Isc group (*P*<0.05).

Additionally, CAT activity was significantly reduced after ischemia compared with the sham group (*P*<0.01). Nevertheless, compared with the Isc group, treatment with gallic acid or GSK650394 alone did not change its activity significantly (Figure 1B). But, coadministration of gallic acid and GSK650394 improved the CAT activity significantly compared with Isc and Isc+G groups (*P*<0.01, *P*<0.05).

Measurement of the activity of GPx indicated that its activity was significantly reduced in the ischemia group (*P*<0.001). However, administration of gallic acid or GSK650394 alone did not change its activity significantly compared with the Isc group (Figure 1C). But, concurrent administration of GSK650394 and gallic acid increased its activity significantly compared with Isc and Isc+G groups (*P*<0.05).

***MDA measurement***

**Figure 2:**
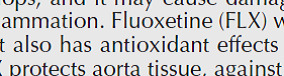
The effects of Gallic acid (G, 30 mg/kg/daily by gavage) and GSK (GSK650394, 1μmol) or their combination on Malondialdehyde (MDA) (A) and total antioxidant capacity (TAC) (B) levels in the heart tissue (mean ± SD, n=10) following heart ischemia/reperfusion injury. ### *P*< 0.001 vs. sham. *** *P*<0.001vs. Isc (ischemic). $$$ *P*<0.001 vs. Isc+G+GSK (One way ANOVA followed by Tukey’s *post hoc* test)

Measurement of MDA concentration indicated that it was significantly enhanced in the Isc and Isc+G groups compared with the sham group (*P*<0.001). However, in those groups treated with GSK650394 alone or in combination with gallic acid, the MDA concentration reduced significantly compared with the untreated ischemic and Isc+G groups (*P*<0.001, Figure 2A).

***TAC assay***

I/R injury significantly decreased TAC in cardiac tissue (*P*<0.001). However, administration of gallic acid or GSK650394 alone did not change its concentration significantly compared with the Isc group (Figure 2B). But, coadministration of both drugs improved the TAC significantly compared with the Isc and Isc+G groups (*P*<0.001).

***Intracellular ROS assay***

**Figure 3:**
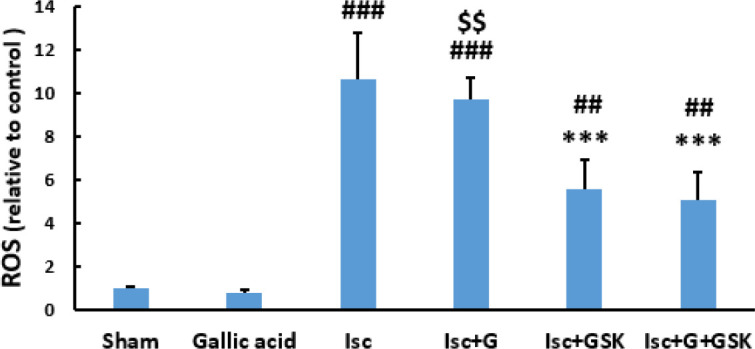
The effects of Gallic acid (G, 30 mg/kg/daily by gavage) and GSK (GSK650394, 1 μmol) or their combination on reactive oxygen species (ROS) in cell sample in the heart tissue (mean ± SD, n=10) following heart ischemia/reperfusion injury. ## *P*<0.01, and ###*P*<0.001, vs. sham. *** *P*<0.001 vs. Isc (ischemic). $$ *P*<0.01, vs. Isc+G+GSK (One way ANOVA followed by Tukey’s *post hoc* test)

Measurement of intracellular ROS showed a significant increase in the Isc group (*P*<0.001). However, treatment with gallic acid alone did not affect its level (Figure 3). But, treatment with GSK650394 alone or together with gallic acid significantly decreased the level of ROS compared with the ischemic group (*P*<0.001). Furthermore, simultaneous treatment with both drugs had more effects than single-drug treatment.

***Cardiac marker enzymes activity***

**Figure 4:**
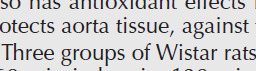
The effects of Gallic acid (G, 30 mg/kg/daily by gavage) and GSK (GSK650394, 1 μmol) or their combination on markers of myocardial damage (mean ± SD, n=10) following heart ischemia/reperfusion injury. # *P*<0.05, ## *P*<0.01, and ### *P*<0.001, vs. sham. ** *P*<0.01, and ***P<0.001, vs. Isc (ischemic). $$ *P*<0.01, and $$$ *P*<0.001, vs. Isc+G+GSK (One way ANOVA followed by Tukey’s *post hoc* test). CK˗MB; creatine kinase˗MB, LDH; lactate dehydrogenase, cTn-I; cardiac troponin-I

Measurement of creatine kinase˗MB activity (CK˗MB) 10 min after reperfusion in the perfusate of the Isc group indicated a significant increase in the enzyme activity (*P*<0.001). However, compared with the Isc group, treatment with gallic acid or GSK650394 alone did not change the enzyme activity significantly (Figure 4A). Nevertheless, coadministration of GSK650394 and gallic acid decreased its activity significantly compared with Isc and Isc+G groups (*P*<0.001 and *P*<0.01, respectively). 

Regarding LDH activity, 10 min after reperfusion, its activity was increased significantly compared with the sham group (*P*<0.001). However, compared with the Isc group, the administration of gallic acid alone did not change the enzyme activity significantly (Figure 4B). In addition, administration of GSK650394 alone or in combination with gallic acid decreased the LDH activity significantly, compared with Isc and Isc+G groups (*P*<0.001).

On the other hand, Troponin-I activity was significantly enhanced in the Isc group compared with the sham group (*P*<0.001) 10 min after reperfusion. But its activity did not change significantly after administration of gallic acid or GSK650394 alone, although coadministration of both drugs decreased the Troponin-I activity significantly compared with Isc and Isc+G groups (*P*<0.01, Figure 4C). 

***Infarct size measurement***

**Figure 5:**
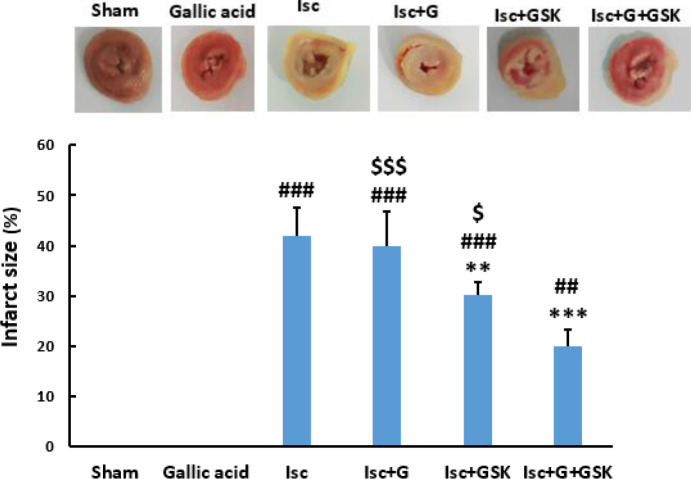
The effects of Gallic acid (G, 30 mg/kg/daily by gavage) and GSK (GSK650394, 1 μmol) or their combination on myocardial infarct size (mean ± SD, n=10) following heart ischemia/reperfusion injury. ## *P*<0.01, and ### *P*<0.001, vs. sham. ** *P*<0.01, and ***P<0.001, vs. Isc (ischemic). $*P*<0.05, and $$$ *P*<0.001, vs. Isc+G+GSK (One way ANOVA followed by Tukey’s *post hoc* test)

The percentage of infarct size was significantly enhanced in the ischemic group compared with the sham group (*P*<0.001). However, compared with the Isc group, treatment with gallic acid alone did not change the infarct volume significantly. But, treatment with GSK650394 alone or together with gallic acid significantly reduced the infarct volume compared with the Isc and Isc+G groups (*P*<0.001). However, the concomitant therapy was more significant than the treatment with GSK650394 alone (*P*<0.05, Figure 5).

***SGK1 gene expression analysis***

**Figure 6:**
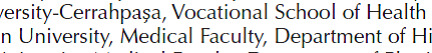
The effects of Gallic acid (G, 30 mg/kg/daily by gavage) and GSK (GSK650394, 1 μmol) or their combination on SGK1 gene expression (mean ± SD, n=10) following heart ischemia/reperfusion injury. # *P*<0.05, and ### *P*<0.001, vs. sham. ***P<0.001 vs. Isc (ischemic). $$ *P*<0.01 vs. Isc+G+GSK (One way ANOVA followed by Tukey’s *post hoc* test)

The results of RT˗PCR indicated that the SGK1 gene expression was significantly enhanced in the ischemic group compared with the sham group (*P*<0.001). However, treatment with gallic acid or GSK650394 alone or the concomitant therapy with both drugs reduced SGK1 gene expression significantly, compared with the Isc group (*P*<0.001). Nevertheless, treatment with both agents at the same time was significantly more effective than treatment with a single agent (*P*<0.01, Figure 6).

## Discussion

Ischemia-reperfusion has had destructive effects on heart function and disrupts the anti-oxidant balance (4). During the cardiac I/R, the increase in ROS level is accompanied by a depletion in SOD and GPx and a decrease in TAC (28), which issue our results confirmed . The results of the present study showed that the activity of anti-oxidant factors such as SOD, GPx, and CAT was decreased significantly in ischemic groups. Subsequently, combined treatment with gallic acid and GSK650394 improved the anti-oxidant factors. Furthermore, simultaneous administration of gallic acid and GSK650394 had better beneficial effects than using them alone. The combination of these two drugs improved anti-oxidant enzyme activity and prevented an excessive increase of intracellular ROS. 

Consistent with this study, many other studies have shown that gallic acid as a potent natural anti-oxidant substance has a useful protective effect against oxidative stress (29, 30). On the other hand, SGK1 is a kinase that may be involved in I/R injury and myocardial infarction (8, 9). Furthermore, SGK1 has been reported to have an essential pressure-related role in the regulation of inflammatory response and cell fate in reperfused-ischemic myocardium (20). The previous opinions suggest that SGK activity aggravates stroke injury and that SGK inhibitors such as EMD638683 and GSK650394, are perhaps valuable candidates for therapeutic intervention (14).

In line with these studies, our data indicated that the activities of GPx, CAT, and SOD enzymes were significantly enhanced by the simultaneous treatment of GSK650394 and gallic acid and increased the detoxification of free radicals. As the results of this research indicated, the activity of anti-oxidant factors and TAC in the heart was significantly restored by treatment with gallic acid and the SGK1 inhibitor. In addition, the present study indicated that a combination of SGK1 inhibitor (GSK650394) and gallic acid had more beneficial effects against myocardial injury than each one alone. 

The TAC test is designed to assay factors of the anti-oxidant protection machine and their capacity to neutralize oxidative pressure (31). In this work, the TAC level analyzed in cardiac tissue was signiﬁcantly lower in the infarcted tissue than in normal tissue and lower in the Isc group compared with the treated groups with coadministration of gallic acid and GSK650394. 

On the other hand, MDA is a crucial biomarker of oxidative pressure, specifically lipid peroxidation (32). Because MDA content is associated with acute myocardial infarction intensity, this index has been used as a determinant of extreme coronary heart disease (33). The present research also showed that treated with GSK650394 alone or in combination with gallic acid, the MDA concentration reduced significantly in ischemic hearts. Meanwhile, based on the level of MDA in the heart tissue, it can be concluded that the simultaneous administration of gallic acid and GSK650394 is beneficial for hearts undergoing I/R injury. Moreover, the previous study has shown that gallic acid can ameliorate anti-oxidant status by inhibiting lipid peroxidation and that it protects cardiomyocytes and lysosomal membranes against isoprenaline-induced oxidative pressure in rats (34, 35). Our results also demonstrated that the myocardial MDA values ​​as a marker of oxidative damage were significantly decreased in the co-administration of gallic acid and GSK650394 compared with administration of GSK650394 alone, indicating that gallic acid inhibited the oxidative stress and lipid peroxidation caused by I/R damage.

Regarding the relationship between I/R injury and oxidative stress, it was shown that the repair of blood flow to the ischemic area leads to the production of large amounts of ROS, causing rapid and intense damage to biomolecules, as the occurrence of known myocardial reperfusion damage (4). The main sources of this ROS are the major contribution of uncoupled eNOS, xanthine oxidase, NADPH oxidases, and mitochondrion (36). Some experiments using animal models indicated that anti-oxidant agents can reduce this phenomenon (5, 16, 17). Free radicals generated by oxidative pressure can trigger DNA strand breaks, lipid peroxidation, and oxidize proteins that may be involved in inflammatory responses and lead to serious tissue injury (37). However, among the relatively large number of polyphenols, various *in vitro* and *in vivo* research (16, 17) indicated that gallic acid is a strong anti-oxidant agent (38). Inconsistent with previous findings, our results indicated that treatment with gallic acid together with GSK650394 significantly reduced the level of ROS in the ischemic group, during which the electrical activity and contractile strength of the heart were improved.

According to the results of this work, cardiac I/R injury caused oxidative damage in the heart tissue, which can be shown through elevation of LDH, cTn-I, and CK˗MB activities, in the ischemic groups’ hearts. In addition, biomarker indices, including CK˗MB, cTn-I, LDH, and activity, released into the blood from damaged tissues, were examined to determine the value of further injury (39). In this research, and consistent with previous reports (5, 26) ischemia, followed by reperfusion, elevated CK˗MB, LDH, and cTn-I concentrations in the coronary effluent 10 min after reperfusion. The results also shows that gallic acid and the SGK1 inhibitor are effective in reducing enzymes’ levels after I/R, and the effects of their combination are more significant. This means that they have a greater cardio-protective effect against myocardial damage. 

On the other hand, previous research has shown that enhanced levels of CK˗MB are strongly correlated with infarct size (16, 26). The size of the infarction is considered the gold standard in assessing the intensity of heart injury following I/R trauma (40). The results of a previous study showed that the use of SGK1 inhibitors, including GSK650394 and EMD638683, significantly reduced the size of the cerebral infarct, with GSK650394 providing greater improvement in infarct area (14). The present data also showed that the infarct volume induced by I/R injury was significantly reduced by treatment with GSK650394 alone or together with gallic acid. As their co-administration had more protective effects, the co-administration of GSK650394 and gallic acid could have more cardioprotective effects against I/R injury than either treatment alone.

Oxidative stress triggers many of the cellular responses that are characteristic of heart failure. These alterations include alteration in gene expression, cellular hypertrophy, and cell death (41). The current findings indicated that the expression of SGK1 was enhanced by I/R injury and reduced by gallic acid and GSK650394. SGK1 is a downstream effector of the phosphoinositide-3 kinase cascade (42) and its expression is highly variable and regulated by a wide variety of triggers (43). A previous study indicated that in cardiac I/R trauma, SGK1 stimulates the release of pro-inflammatory factors and decreases the level of anti-inflammatory factors, but inhibition of this enzyme reduced proinflammatory cytokine in cardiac I/R (20). These results are consistent with our findings in the model of heart I/R damage, and our data also suggest a key role for SGK1 in the regulation of I/R damage. Likewise, our data showed that treatment with gallic acid or GSK650394 alone or the combination of both drugs significantly reduced SGK1 gene expression. However, simultaneous treatment with both drugs had more significant effects than single-drug treatment. 

**Table 1:** Groups of rats that have been used in this study

Groups*	**Pretreated with gallic acid (30 mg/kg) or vehicle (Saline) for 10 days**	**GSK650394 (µmol) perfused for 5 min before Ischemia induction**
Sham	Saline	0
Gallic acid	Gallic acid	0
Isc**	Saline	0
Isc+G	Gallic acid	0
Isc+GSK	Saline	1
Isc+G+GSK	Gallic acid	1

*, 10 rats in each group; **, Ischemic/reperfusion groups

## Conclusion

Briefly, the present study demonstrated that gallic acid and SGK1 inhibitors can act as potential protective agents in a rat model of cardiac I/R injury. In addition to improving heart function, they also improved biomarkers of heart injury and reduced oxidative stress. However, as far as we know, the combination of these two drugs has not been used concomitantly to investigate their combination effect. The current study showed for the first time that concomitant administration of gallic acid and GSK650394 as an SGK1 inhibitor can potentiate each other’s effects and their combination affects I/R injury complications more significantly than each one alone. Therefore, it can be suggested to consider the simultaneous administration of such drugs to treat or prevent the effects of cardiac complications induced by ischemia/reperfusion injury.

## Authors’ Contributions

All authors contributed to the study’s conception and design. FS, MD, AM, AS, and MB performed material preparation, data collection, and analysis . FS and MB wrote the first draft of the manuscript and all authors studied and commented on previous versions of the manuscript. All authors approved the final manuscript.

## Funding

This research project was sponsored by the Research Vice-Chancellor of Ahvaz Jundishapur University of Medical Sciences (AJUMS) and the Persian Gulf Physiology Research Center (Grant no. APRC-0003).

## Ethical Approval

The protocols of the experiments were approved by the research ethics committee of the Research Center & Experimental Animal House at Ahvaz Jundishapur University of Medical Sciences according to the ethical principles and national norms and standards for conducting Medical Research in Iran (ID: IR.AJUMS.ABHC.REC.1400.032, dated: 2021-05-25).

## Conflicts of Interest

The authors have no relevant financial or non-financial interests to disclose.
